# Cadmium Exposure as a Putative Risk Factor for the Development of Pancreatic Cancer: Three Different Lines of Evidence

**DOI:** 10.1155/2017/1981837

**Published:** 2017-11-16

**Authors:** Aleksandra Buha, David Wallace, Vesna Matovic, Amie Schweitzer, Branislav Oluic, Dusan Micic, Vladimir Djordjevic

**Affiliations:** ^1^Department of Toxicology “Akademik Danilo Soldatović”, University of Belgrade, Faculty of Pharmacy, Belgrade, Serbia; ^2^School of Biomedical Science, Oklahoma State University Center for Health Sciences, Tulsa, OK, USA; ^3^Emergency Center, Clinical Center of Serbia, Belgrade, Serbia; ^4^First Surgical Clinic, Clinical Center of Serbia, Belgrade, Serbia

## Abstract

Although profoundly studied, etiology of pancreatic cancer (PC) is still rather scant. Exposure to cadmium (Cd), a ubiquitous metal associated with well-established toxic and carcinogenic properties, has been hypothesized to one putative cause of PC. Hence, we analyzed recently published observational studies, meta-analyses, and experimental animal and in vitro studies with the aim of summarizing the evidence of Cd involvement in PC development and describing the possible mechanisms. Consolidation of epidemiological data on PC and exposure to Cd indicated a significant association with an elevated risk of PC among general population exposed to Cd. Cadmium exposure of laboratory animals was showed to cause PC supporting the findings suggested by human studies. The concordance with human and animal studies is buttressed by in vitro studies, although in vitro data interpretation is problematic. In most instances, only significant effects are reported, and the concentrations of Cd are excessive, which would skew interpretation. Previous reports suggest that oxidative stress, apoptotic changes, and DNA cross-linking and hypermethylation are involved in Cd-mediated carcinogenesis. Undoubtedly, a significant amount of work is still needed to achieve a better understanding of the Cd involvement in pancreatic cancer which could facilitate prevention, diagnosis, and therapy of this fatal disease.

## 1. Introduction

Pancreatic cancer (PC) is one of the most lethal human cancers and an important cause of cancer-associated-mortalities worldwide [1, 2]. With no specific symptoms observed until in its advanced and incurable stage, PC develops insidiously and at the time of diagnosis majority of patients already faces an advanced form of the disease. Thus, PC has the worst survival rate of any major cancer, and its mortality has been gradually increasing over the past decades. Profound studies have been undertaken with the aim of improving our understanding of this aggressive disease, and while certain advances in molecular biology have greatly enhanced the understanding of PC pathogenesis, the etiology remains rather elusive. Some genetic risk factors have been identified, such as mutations of K-ras oncogene and various suppressor genes but they account for less than 10% of all PC causes [3]. The main nongenetic environmental factors that have been associated with PC initiation so far are inhalation of cigarette smoke, exposure to mutagenic nitrosamines, chlorinated hydrocarbon solvents, and heavy metals [4].

Among different heavy metals, attention must be given to involuntary human exposure to cadmium (Cd). This toxic element is currently one of the most abundant occupational and environmental pollutants; it is present in the diet, tobacco smoking, air, soil, and water [5]. Although the production of Cd has declined, its persistence and long biological half-life have led to the increase of health risks this metal impose [5]. Numerous studies have confirmed its toxic effects on many organs systems and this toxicity is dependent on dose, route, and duration of exposure. Cd has been shown to produce damage to the lung, liver, kidney, bones, and testes [5–7]. Novel investigations point to its endocrine disrupting properties suggesting Cd role in diabetes mellitus [8], different types of thyroid disruption [9, 10], and its estrogenic activity [11, 12]. Cadmium and its compounds have been classified as known human carcinogens by the International Agency for Research on Cancer since 1993 [13] based on epidemiological studies showing a causal connection with the development of lung cancer. Epidemiological studies have also implicated Cd connection to kidney cancer [14–16], prostate cancer [15, 17, 18], cancer of testis [18], bladder cancer [19], breast cancer [20], and overall cancer mortality [21].

The link between Cd exposure and PC development has been extensively investigated as well, especially since the pancreas is considered as an important organ for Cd accumulation [22, 23]. Furthermore, many of known and well-established risk factors of PC are connected to an increased Cd body burden, that is, age and smoking. This review will summarize, evaluate, and discuss information on Cd role in PC development collected from available and recently published observational studies, meta-analyses, experimental animal, and in vitro studies.

## 2. Cd Role in PC Development-Human Studies

First authors to hypothesize that Cd exposure can cause PC development were Schwartz and Reis [24]. They conducted a meta-analysis of reports on the mortality of workers exposed to Cd. Their hypothesis that exposure to Cd increases the risk of death from PC was supported by the evidence collected from three cohort studies included in this meta-analysis [25–27]. The same year, Ojajärvi et al. [28] conducted meta-analyses aiming to consolidate epidemiological data on pancreatic cancer and various worksite exposures. This study, however, did not provide evidence on Cd pancreatic carcinogenicity.

In next few years, studies in population without occupational exposure history, that is, general population, were conducted with the aim of collecting epidemiological evidence that Cd is a potential human pancreatic carcinogen. Li et al. [29] carried out a prospective study in a general Japanese population between 45 and 74 years of age. Dietary Cd intake was estimated through questionnaires and during next nine years cancer development was followed. There was no evidence of an association between cancer development and Cd intake in this study. Analysis of data collected from Third National Health and Nutritional Examination Survey (NHANES III) cohort showed an association between Cd exposure, independent of cigarette smoking, and mortality from PC, but the Cd-cancer association was only observed in males [30]. These results add to evidence of Cd exposure as a possible cause of PC is independent of smoking. In a case-control study conducted in Spain to determine the relationship between PC risk and trace elements levels, toenail samples of PC cases and hospital controls were used to determine levels of different trace elements among other Cd. The results of the study confirmed Cd role in pancreatic cancer risk [31]. Luckett et al. [32] showed evidence of an etiological link between Cd exposure and PC in the population of South Louisiana, USA, which has the history of PC that exceeded the national average. The Cajun population, especially the older cohort investigated in this study, consume large amounts of seafood, rice, and pork which are important sources of Cd, possibly increasing their exposure to Cd. One mechanism through which Cd could promote PC increased synthesis of metallothionein since positive staining for metallothionein in pancreatic carcinomas was often associated with worse histological grade of carcinoma, liver metastases, and shorter survival as shown by Ohshio et al. [33] in a sample of 75 pancreatic duct cell carcinomas. Environmental exposure to Cd among inhabitants living in the Cd-polluted Kakehashi River basin in Japan showed no significant increase for malignant neoplasm mortalities (including PC). Garcia-Esquinas et al. [34] examined the association between the long-term Cd exposure as measured in urine and cancer mortality in Native Americans participating in the Strong Heart Study (a prospective cohort study with a follow-up of 20 years). The authors found prospective association between low-to-moderate Cd exposure and total cancer mortality and mortality from lung and PC. Similar to Adams et al. [30], after adjusting for smoking status, authors account Cd, an independent risk factor for PC. In a meta-analysis that included the studies listed above [29–32, 34, 35], the authors found a positive association between Cd exposure and the increased risk of PC in males, while no significance was observed in females [36]. This phenomenon was clarified with the hypothesis that steroid hormones can have a protective role in the development of pancreatic cancer and that women tend to consume more vegetables and fruits, that is, more antioxidants and fiber [37, 38]. One more study attempted to establish a correlation between environmental exposure to Cd and PC development. In a pilot study, Kriegel et al. [39] tested the hypothesis that PC patients in East Nile Delta region of Egypt (a region with high incidence of early-onset pancreatic patients) were exposed to higher levels of Cd than the subjects with no PC diagnosis. A significant association between PC and serum Cd levels was confirmed, once again proving Cd as an independent risk factor for PC.

Summarized cohort and case-control observational studies represent an expanding body of evidence that Cd is a risk factor for PC development even as a factor independent of smoking and occupational exposure, particularly in man. Nevertheless, Cd exact role in PC carcinogenesis is still unknown. Our recent investigations pointed to the direct role of Cd in PC development. Namely, in our pilot case-control study, concentrations of Cd were significantly higher in human PC tissue than in control pancreatic tissue [40]. Furthermore, tissue concentrations of Cd in tissue surrounding tumor were still significantly higher than in control tissue [41]. However, understanding the role of Cd in the PC development is possible only through additional work that will provide solid evidence of this association under the experimental conditions and give better insight into cellular and molecular changes that are happening in pancreatic cells under the Cd influence.

## 3. Cd Role in PC Development-Animal Studies

Cadmium is a complete carcinogen in experimental animals; it has proven to effectively induce cancers at multiple sites, by various routes of exposure and doses. Inhalation in rats induces pulmonary adenocarcinomas, ingestion or injection can induce tumors and preneoplastic lesions within the rat prostate, and at the site of subcutaneous (s.c.) or intramuscular (i.m.) injections Cd can produce local mesenchymal tumors. Other targets of cadmium in rodents include the liver, adrenal, pituitary glands, hematopoietic system, and pancreas [14, 42, 43].

Carcinogenic effects of Cd in the animal pancreas are very complex. As reviewed by Waalkes et al. [44], Cd impact on the incidence of tumors in the rat pancreas was dependent on the conditions of exposure. When Cd is administrated via s.c. route, it induces a dose-related reduction in pancreatic tumor formation in Wistar rats. When given via s.c. injection, but concurrently with calcium-induced islet cell tumors of the rat, multiple s.c. injections of Cd in Wistar and Fischer rats resulted in transdifferentiation of pancreatic cells into hepatocytes. In fact, Cd is one of the most potent agents known to induce transdifferentiation, that is, metaplasia of the pancreatic cells [44, 45], and since the process of metaplasia involves cellular dedifferentiation, proliferation, and redifferentiation, it may increase the risk for neoplasia [46].

Due to lack of an apparent biological mechanism for excretion, Cd accumulates in tissues, with the largest amounts deposited in kidneys (half-life in the cortex is 10 to 30 years) and liver, followed by the pancreas and lungs [5]. Our recent findings also confirmed Cd deposition in the pancreas, as single oral doses of 15 and 30 mg Cd/kg b.w. produced significantly higher levels of Cd in pancreatic tissue of Wistar rats when compared to untreated controls 24 h after administration (unpublished data). Furthermore, oral doses of 5 mg CdCl_2_/kg b.w. given over 4 weeks resulted in increased Cd concentrations in the pancreas of rats when compared to controls [47].

Potential mechanisms of Cd carcinogenicity in animals have been suggested by Schwartz and Reis [24]. According to the authors, substitution of Cd with zinc (Zn) may be a central mechanism underlying Cd carcinogenicity. Zn is known to be essential for DNA, RNA, and protein synthesis, that is, for cell division, and pancreas contains high levels of Zn. Evidence of Cd effects on Zn homeostasis was reviewed by Matović et al. [6] and Brzóska and Moniuszko-Jakoniuk [48]. Recently, we demonstrated these Cd interactions with Zn in the pancreas of rabbits [49, 50]. Further illustration of the importance of Cd-Zn interactions in PC development is that the effects of Cd on several organs [51] and even tumor formation [52] can be suppressed by the simultaneous Zn treatment. Another possible mechanism of Cd carcinogenicity assumed by the same authors [24] is the ability of Cd to induce mitogenesis in pancreatic cells as reflected by the increased metallothionein synthesis to which Cd is highly bound in the pancreas of rats.

Other studies conducted on animals revealed different toxic effects of Cd on pancreas and mechanisms of these effects that could explain Cd carcinogenicity in the pancreas. One well-established general mechanism of Cd toxicity is oxidative stress induction as reviewed in our recent papers [6, 7]. Cadmium ability to produce oxidative stress was shown in the pancreas of rats treated with 5 mg CdCl_2_/kg b.w. during 4 weeks [47]. Levels of reactive oxygen species and lipid peroxidation in the pancreas of Cd-intoxicated animals were significantly higher than in controls, while levels of various antioxidant enzymes were significantly decreased as compared to those of the controls, suggesting cellular oxidative stress-mediated toxicity. In another study conducted on mice, an interesting mechanism by which Cd produces pancreatic toxicity and potential carcinogenicity was introduced. A single s.c. injection of 1 mg Cd/kg to mice had no obvious toxic effects on pancreas at either 1 or 5 days after Cd treatment. Withal, the activities of pancreatic proteases trypsin, chymotrypsin, and carboxypeptidase A were significantly decreased at 1 day after treatment, while the activity of carboxypeptidase B was not changed. These changes in enzymatic activity returned to control levels 5 days after Cd treatment, which suggests that Cd-mediated effects could be reversible. Bearing in mind the complex roles of these enzymes within the organism, the inhibition of these enzymes may have an impact on various critical physiological functions, among which cellular transdifferentiations in pancreas and cancers can be assumed [53].

From in vivo studies, both in humans and in rodents, there is evidence that suggests the role of Cd in the development of PC through cellular changes involving oxidative stress, changes in apoptotic pathways, and epigenetic changes. Human studies tend to involve post hoc observation and the description of Cd-mediated toxicity in the pancreas [54], renal tubule [55], and liver [56]. Human studies have been extended to include nonhuman species, such as mouse and livestock [53, 57]. Many of these organ deficits have been attributed to the actions of Cd and its ability to increase the generation of free radicals, alter cellular apoptosis [54], and promote epigenetic changes [58]. Collectively, it is apparent that in vitro studies will be needed to augment findings in vivo. The use of in vitro analysis will permit investigators to probe the cellular changes that occur following Cd exposure, which lead to altered pancreatic function and the development of PC.

## 4. Cd Role in PC Development-In Vitro Studies

An important tool for studying the carcinogenic actions of Cd is the development of an appropriate in vitro model [59, 60]. Utilizing a combination of in vivo and in vitro data, other investigators have attempted to describe the carcinogenic action of Cd from an intracellular perspective [15, 61]. To date, data from in vitro studies have been difficult to interpret. Many investigators report only the positive effects following Cd exposure while using excessive concentrations of Cd, which would skew data interpretation [43]. There have been excellent reviews that focused on the generalized role of Cd as a carcinogenic agent [24, 42, 43, 62]. Model systems for the study of Cd-mediated carcinogenic effects are limited, and model systems for the study of PC are even fewer. Multiple studies have linked Cd-mediated effects to the development of PC or hypothesized that Cd could be involved in PC development [15, 24, 31, 32, 39, 40, 63]. Human studies have shown that Cd concentrations are approximately 5 times higher in tissue that is obtained directly from a pancreatic tumor when compared to Cd concentrations found in control/non-PC tissue [40]. Even tissue near the tumor displayed significantly higher Cd concentrations when compared to control tissue [40]. In vitro studies which substantiate human in vivo studies have been equivocal in their findings. From the current literature, Cd-mediated effects may be via (1) reactive oxygen species (ROS)/free radicals or oxidative stress, (2) changes in apoptotic pathways, (3) epigenetic changes, or (4) a combination of effects via multiple pathways. Collectively, it is clear that delineating the mechanisms of Cd in promoting the development of PC will be time-consuming.

Oxidative stress or the generation of reactive oxygen species (ROS, free radicals) appears to have an impact on the development of PC or generalized damage to the pancreas. There have been relatively few studies associated with Cd-induced oxidative stress in the pancreas. There have been numerous reports demonstrating Cd bioaccumulation in the pancreas [24, 31, 32, 34, 39, 40, 42, 43, 59]. Cadmium is considered a redox-inactive cation, and its primary mode of toxicity is through zinc finger or sulfhydryl binding [64]. Inflammation following oxidative stress, a precursor for potential tumor development, is an underlying cause of pancreatitis [65–67]. There are many reports and reviews demonstrating ROS-mediated toxicity associated with Cd exposure [68, 69]. Studies showing Cd-mediated oxidative stress have utilized multiple cells lines or in vitro model systems [70–72]. Determination of the specificity of Cd-mediated cellular damage requires addressing the question of whether the effects are direct or indirect. Exposure to particular environmental pollutants will induce oxidative stress, and the generation of free radicals will kill some cells, but in the surviving cells, apoptosis will be initiated to attempt cellular repair [73]. In neuronal cultures, Cd induces the formation of free radicals which result in cellular damage but also trigger the activation of the mTOR pathway leading to cellular death [69]. The neuronal culture findings are supported by at least one study in pancreatic *β*-cells that suggests that Cd-mediated damage to *β*-cells is initiated by oxidative stress which reduces the density of *β*-cells, followed by mitochondrial-induced initiation of apoptosis [74]. When all of the evidence is taken together, it does appear that oxidative stress is involved in the development of PC. This involvement may be via an indirect mechanism, such as causing the inflammation which leads to the development of PC following the release of inflammatory mediators. Additional work needs to be performed to further our understanding of the molecular and intracellular actions of Cd.

The regulation or dysregulation of apoptotic pathways by Cd is particularly intriguing. Cd may elicit indirect actions on apoptosis (as through oxidative stress discussed previously) or through direct actions on apoptotic regulatory proteins [75]. Like mercury, Cd in its divalent form has been shown to interact with zinc-binding sites and thiol groups that are commonly found in proteins [15]. Interestingly, Cd-mediated effects on apoptotic pathways are as unclear as what is observed for Cd-mediated oxidative stress. The cell type that is exposed to Cd seems to be an important factor [61]. A previous study demonstrated Cd-induced apoptotic in pancreatic *β*-cells that were secondary to oxidative stress [74]. The process of normal apoptosis is necessary to maintain the integrity of the cells. During apoptosis, repairs are attempted on damaged cells, and if the damage is irreparable, the cells are eliminated maintaining cellular integrity. If cell growth is permitted unfettered, an increased number of mutated/damaged cells will be allowed to proliferate. Conversely, if cell growth is reduced to an excessive number of cells entering apoptosis, this too will be detrimental to cellular integrity [43, 62]. One mechanism for Cd-induced apoptotic inhibition is through suppression of caspase activity. This hypothesis is supported by data that suggest either caspase-3 or caspase-7 inhibition following Cd exposure [46]. Inhibition of caspase would reduce the number of cells in apoptosis resulting in an increased proliferation of defective cells. The tumor suppressor protein, p53, has been a target of investigation by numerous laboratories. The results for Cd-mediated alterations in p53 activity have not been clear. In prostate cells, Cd induces p53 activity, which would suggest tumor suppression [76]. Resistance will develop to elevated p53 activity resulting in tolerance to the tumor-suppressive activity of p53. Contrary to prostate cell data, Cd decreases p53 activity and increases cellular proliferation in zebrafish liver cells [75]. Increased activation of tumor-promoting oncogenes will lead to an increased risk for PC [77]. Cadmium exposure reported can increase the activity of the K-ras oncogene, leading to increased tumor formation [78]. In immortalized control pancreatic cells [HPNE (Human Pancreatic epithelial Nestin-Expressing)], K-ras activation increased signaling in PI3K/Akt and ERK1/2 pathways as well as TGF*α* signaling and is involved in PC formation [79]. Also, p38/MAPK pathway activation is involved in observed p16 upregulation which also increases [79]. Cadmium has been reported to interact with Zn, interfering with DNA repair proteins [15, 62, 76, 80]. Molecular changes in the 3-dimensions structure of the apoptotic proteins will affect the cells ability to repair cellular injury which will result in uncontrolled cellular growth that perpetuates the damage associated with the modified cells. It is apparent that Cd-mediated effects on apoptosis are highly dependent on the (1) type of exposure: in vitro versus in vivo, (2) cell or organ type, (3) gender, (4) Cd concentration, and (5) the point in the apoptotic pathway being investigated. Therefore, caution is needed in planning in vitro studies examining Cd-mediated changes in apoptosis.

Epigenesis, as it relates to genetic changes (epigenetics), is defined as gene alterations in response to influences, such as environmental/occupational insults which could impact the expression of genetic material in either a positive or negative way. To date, no studies have intensively studied the epigenetic role of Cd in the development of PC. Prenatal exposure to Cd during embryogenesis appears to involve changes in DNA methylation [81, 82]. The authors acknowledge that many of the existing studies have extrapolated epigenetic changes following exposure to high concentrations of Cd and that these interpretations may be skewed [81]. An increasing number of studies have examined the effects of various pollutants, including Cd, on epigenetic changes in adults [83–86]. Multiple investigators have reported that Cd is a carcinogen, but weakly mutagenic [43, 61, 84, 85]. The current theory suggests that Cd can act epigenetically through several molecular mechanisms: (1) interruption of DNMT activity, (2) interference with DNA repair mechanism, or (3) miRNA upregulation. Studies have shown plasticity in cadmium reactivity by demonstrating its initial association with global hypomethylation at several promoter sites, followed by consequent hypermethylation, indicating restored viability after 8 hours of exposure [87]. Martinez-Zamudio and Ha [85] have examined the literature to determine whether or not Cd-mediated epigenetic changes could be inheritable. The results were inconclusive and suggested that more work would be needed to better understand the inheritable potential of genetic changes following Cd exposure. In lung fibroblast cells, Cd exposure resulted in an increased in DNA methylation of p16 and an upregulation of DNMT3b [84]. The duration of exposure to Cd seems to significantly affect the methylation process, with acute Cd exposure resulting in hypomethylation, followed by hypermethylation after chronic exposure [82, 85]. Considering the long biological half-life exhibited by Cd, even acute exposures would be “chronic” exposure due to the extended duration of the body burden. In vitro Cd exposure has been shown to increase marker expression related to the conversion of noncancer stem cell for cancer stem cell populations in both breast and liver cancer cell lines [83]. Increased levels of p-Ras, p-Raf-1, p-MEK-1, and p-ERK-1 protein were observed in suggesting an increase in the Ras signaling cascade [83]. The c-fos protooncogene is upregulated by Cd exposure and consequently interferes and suppresses the p53 tumor suppression mechanism [24]. Cd's ability to replace zinc in the zinc finger structure of xeroderma pigmentosum A protein prohibits cells from removing lesions on the DNA helix, blocking transcription, and translation [80]. It is also possible that Cd recruits transcription factors usually recruited by zinc, increasing mitogenesis and propagating the replication of damaged DNA [43]. The final potential epigenetic mechanism influenced by cadmium exposure manifests in increased miRNA production. Aberrant expression of miRNA fragments results in epigenetic transcriptional regulation. For example, recent studies have indicated an upregulation of miRNA 146 expression in steel workers exposed to Cd inhalants. miRNA 146 is associated with nuclear factor kappa-B, with links to carcinogenesis and inflammatory response [88]. Recent work has examined the epigenetic role of Cd in the development of various cancers such as melanoma [89], gastrointestinal tract [90], and uterine-endometrial carcinomas [91]. Across each specific cancer, the key epigenetic events that transpired were increased DNA methylation, increased expression, and production of either pro- or antiapoptotic proteins, such as DNMTs, p53, p16, and other proinflammatory proteins [89–91]. Currently, there have been no studies which focused solely on epigenetic changes due to Cd exposure in PC.

Collectively, in vitro studies have yielded a confusing landscape to date. It is apparent that a considerable amount of work is still needed to understand further the cellular and molecular pathways that are altered following Cd exposure. The current literature would suggest that Cd is not directly genotoxic but instead may directly or indirectly increase oxidative stress and the formation of DNA- and protein-damaging free radicals. Cd has also been shown to alter cellular ability to enter apoptosis and perform cellular repair. Changes in apoptosis could also lead to epigenetic changes due to damaged and mutated DNA. In vitro investigation into Cd's role in the development of PC will be a dynamic and evolving area of research for many years to come.

## 5. Summary

Although interest in Cd as a potential carcinogen has risen dramatically in the last two decades, more work must be done to further our understanding of Cd-mediated toxic mechanisms. Mounting evidence suggests that Cd exposure is a major health concern and the ability that Cd displays to bioaccumulate and to remain persistent in the environment for years further compounds the dangers associated with Cd exposure. An increasing number of human and experimental clinical studies are specifically investigating the effects of Cd on the pancreas. Yet, the data is somewhat conflicting, but more evidence points towards Cd as an agent responsible for the development of PC. When the in vivo data is used as a foundation for in vitro studies, there is an increasing body of evidence that outlines the toxic mechanisms associated with Cd exposure. Although multiple mechanisms are proposed, the three predominant toxic mechanisms are (1) alterations in the redox status of the cell and generation of free radicals, (2) changes in the apoptotic pathways necessary for normal cell survival, and (3) epigenetic changes that would interfere with the proper and vital functioning of DNA and RNA. Significantly more work needs to be done to expand our knowledge of the Cd-induced modifications in the pancreas that promote the pathogenesis of PC.
